# The implications of growth mindset for depression, well-being, and adjustment over 2 years during the COVID-19 pandemic

**DOI:** 10.1371/journal.pmen.0000182

**Published:** 2024-12-19

**Authors:** Yena Kyeong, Esra Kürüm, Pamela Sheffler, Leah Ferguson, Elizabeth L. Davis, Carla M. Strickland-Hughes, Rachel Wu

**Affiliations:** 1 Department of Psychology, University of California, Riverside, Riverside, California, United States of America; 2 Department of Statistics, University of California, Riverside, Riverside, California, United States of America; 3 Department of Psychology, University of the Pacific, Stockton, California, United States of America; Hamdard University - Islamabad Campus, PAKISTAN

## Abstract

Growth mindset, the belief that abilities and attributes are changeable, has been implicated in better mental health and health behaviors and may be especially critical during challenging life events. One goal of this prospective longitudinal study was to investigate the role of growth mindset in adults’ mental health (i.e., depression, well-being, and adjustment of daily routines) over two years of the COVID-19 pandemic. We also examined this relationship in older adults who had participated in a prior learning intervention including growth mindset training (compared with those who had not). Adults ages 19 to 89 from ethnically diverse backgrounds in Southern California (*n* = 454) were surveyed at three timepoints between June 2020 and September 2022. In Study 1 focusing on this wide age range (*n* = 393), we found that growth mindset was associated with lower levels of depression and higher levels of well-being and adjustment, after accounting for various sociodemographic factors. Study 2, which focused on older adults (*n* = 174), largely replicated the findings from Study 1. Furthermore, the conducive effect of growth mindset on well-being was marginally greater among those who had participated in the intervention, and those who had participated in the intervention showed an increase in well-being over time, while well-being scores decreased in the control group. Together, our findings suggest that growth mindset may be an important protective factor for mental health during challenging times.

## Introduction

Widespread, long-term physical distancing increased depression rates and lowered well-being among adults around the world [1, 2]. Reflecting that social disconnectedness can confer a risk to mental health problems [3], social isolation and loneliness during the pandemic were found to be associated with higher depression and suicidal ideation and lower well-being among adults [4, 5]. Adapting and adjusting daily tasks, such as using video calls to maintain social interactions, could mitigate the impact of social isolation amidst social distancing restrictions, given that flexibly adapting one’s behaviors is a key to successful adjustment in changing environment [6]. However, given that this “new normal” heavily relies on digital access and literacy, it continues to pose additional risks for less advantaged communities, such as some older adults or ethnic/racial marginalized and minoritized groups [7].

Research on psychological factors contributing to resiliency in mental health is emerging in the aftermath of the COVID-19 pandemic. For example, factors such as mindfulness, optimism, and social support were found to be predictive of lower depressive symptoms [8, 9]. For subjective well-being, having more social networks and less financial burden [10], as well as greater meaning in life [11], have been identified as protective factors amid the pandemic. Although scant research has examined the precursors of adjustment of daily tasks during the pandemic specifically, some evidence suggests that psychological flexibility (i.e., recognizing and adapting to life changes) predicts adaptive coping amidst the COVID-19 pandemic [12, 13]. Growth mindset, the belief that abilities and attributes are changeable [14, 15], may be an important factor related to resiliency in mental health, in particular depression, well-being, and adjustment to challenging life circumstances. According to Dweck’s mindset theory, having a growth mindset is associated with mastery goals, positive affect, and persistence, and those with a higher growth mindset tend to view challenges and setbacks as an opportunity for growth and learning [14–16]. Furthermore, evidence indicates that the implications of a growth mindset about intelligence (i.e., the malleability of intellectual ability) can extend beyond academic outcomes to mental health [17, 18]. Although traditionally studied in children and adolescents’ motivation in the academic arena, researchers posit that this may be a potential contributing factor for successful aging later in life [19], predicting older adults’ better memory performance [20] and healthful behaviors [21].

Notably, the beneficial effect of a growth mindset on mental health might be especially salient in times of challenge, since it predicts one’s reactions toward setbacks [15] and can cultivate resiliency [22]. Growth mindset can shape one’s attributions for setbacks, as well as coping strategies, making individuals with higher growth mindset more resilient when facing challenges [15]. A few prior studies allude that a growth mindset may contribute to better mental health amid the COVID-19 pandemic. For example, one study on Indonesian college students found the link between growth mindset and positive mental health [23]. Another study on Chinese adolescents revealed that growth mindset is predictive of more positive coping strategies, which in turn, predicts better mental health during the pandemic [24].

Because the aforementioned research on growth mindset and mental health outcomes during the pandemic has focused on younger populations, it is unclear whether growth mindset has similar implications for older adults’ mental health. Prior to the pandemic, a study by Bartholomaeus et al. [25] found that an intervention including growth mindset training helped buffer against older adults’ feelings of social isolation. Furthermore, given that adjustment in the pandemic world entails garnering new skills (e.g., technologies, devices) for older adults [26], a learning-related belief, such as growth mindset, may particularly foster the mental health of older adults by facilitating their learning to adapt to new circumstances. During the pandemic, engaging in daily mastery experiences, such as learning or using skills, was found to attenuate the negative effects of increasing social disconnection on psychological well-being [27]. As older adults are less likely to be proficient in new technology compared to younger age groups, which has been implicated in their pandemic-related social exclusion [28], this issue also may affect their ability to adjust their daily tasks. Given the demands of adapting to the “new normal” and the necessity to learn new skills during the pandemic, there is a need for more research attention on the role of motivational factors, such as growth mindset, particularly in older adults.

## The present study

The main goal of this prospective longitudinal study was twofold. First, we investigated the role of growth mindset on mental health over two years of the COVID-19 pandemic among adults in a wide range of age (Study 1). The data from this correlational study consisted of three timepoints over two years. As markers of mental health, we focused on the levels of depression, subjective well-being, and adjustment of daily tasks. We expected that higher participants’ growth mindset would predict lower depression, higher well-being, and a greater likelihood of adjustment to daily tasks. The sample was diverse in terms of demographics, such as race and age. Considering the disproportionate impact of the COVID-19 pandemic on mental health for racially minoritized groups in the United States [29], we also explored the potential effects of sociodemographic variables in addressing the main research question.

Second, focusing on the sample of older adults (Study 2), we investigated the potential role of the prior learning intervention. Although this study was also correlational in nature, participants were divided into two groups based on a condition from a prior experiment study. In this intervention that took place before the pandemic, older adults learned multiple new skills (e.g., Spanish, painting, how to use an iPad) for three months, and they increased their cognitive functioning up to similar levels to middle-aged adults by the end of the intervention [30] and also increased their growth mindset at post-test [31]. Given that such gains may foster psychological well-being in the long run [32], we anticipated that those who engaged in the learning intervention may benefit from an increased growth mindset, leading to better mental health more so than those who did not participate in the intervention.

## Materials and methods

### Ethics statement

The study procedures were reviewed and approved by the institutional review board at the University of California, Riverside. For this study, a waiver of written consent was obtained for the online survey. Upon reviewing the consent form presented on Qualtrics, participants were given the option to select either “Yes, I consent to participate.” or “No, I do not consent.” Those who provided their consent by selecting the former were directed to the survey link.

### Participants and procedure

Data collection initially aimed to recruit participants from three age groups: younger (ages 18 to 39), middle-aged (ages 40 to 59), and older (ages 60 to 90) adults. We performed a priori power analysis using techniques proposed by Cohen [33]. Results indicated that we could achieve a power of 80% to detect small to medium (0.05–0.15) effect size for Cohen’s *f*^2^ with 30 participants per group at alpha 0.05. Considering the potential attrition in a longitudinal design, we aimed to recruit 1.5 times as many participants per age group. Participants in the current study included 454 younger to older adults residing in Southern California who were surveyed at three timepoints between June 2020 and September 2022.

Specifically, at Wave 1, data collection spanned approximately one month (June 12, 2020 to July 21, 2020). Data collection at Wave 2 (March 1, 2021 to April 20, 2021) and Wave 3 (August 9, 2022 to September 23, 2022) similarly spanned around one month each. Across the three timepoints, our initial total sample included 952 datapoints (i.e., *n* = 494 at Wave 1, *n* = 251 at Wave 2, *n* = 207 at Wave 3). Among them, incomplete responses missing all study variables (*n* = 49 at Wave 1, *n* = 16 at Wave 2, *n* = 16 at Wave 3) were removed. There was one participant who was underage (i.e., under the age of 18) when taking the survey, thus their responses at Waves 1 and 2 were removed. Furthermore, four participants at Wave 2 and one participant at Wave 3 took surveys twice, and we removed their later responses. At Wave 3, we employed seven attention-check items and excluded 19 cases who failed to pass 85% of these checks. Consequently, the final total sample retained 454 unique cases, with 845 datapoints (i.e., 444, 230, and 171 participants at each wave, respectively). Among these 454 unique cases, ten participants did not have their data at Wave 1: one participant, whose data at Waves 1 and 2 were excluded due to being underage, and nine participants who took part in our prior learning intervention and missed the first survey.

Among the final total sample, Study 1 utilized younger to older adults (*n* = 393; 73% female) age range from 18 to 89 years old (*M* = 46.45, *SD* = 17.67) at the initial timepoint. In Study 2 which included only the older adult group (*n =* 174; 82% female), participants were from 58 to 89 years old (*M* = 69.99, *SD* = 6.91). In this sample, a subset of older adults (*n* = 61; 89% female) who had participated in a learning intervention before the COVID-19 pandemic [34] was included. Table 1 presents the demographic characteristics of the current sample.

**Table 1:** Demographic characteristics of the study samples.

Variables	Study 1 (*n* = 393)	Study 2 (*n* = 174)
Ethnicity, *N* (%)^1^		
Hispanic or Latino	120 (31)	21 (12)
Not Hispanic or Latino	264 (67)	146 (84)
Prefer not to answer	9 (2)	7 (4)
Race, *N* (%)^2^		
American Indian/Alaska Native	7 (2)	1 (1)
Asian	40 (10)	11 (6)
Native Hawaiian or Other Pacific Islander	3 (1)	–
Black or African American	30 (8)	18 (10)
White	232 (59)	128 (74)
More than one race	28 (7)	11 (6)
Other	37 (9)	4 (2)
Prefer not to answer	16 (4)	1 (1)
Education Level, *N* (%)		
Professional degree (M.D., Ph.D., etc.)	31 (8)	13 (7)
Master’s degree (M.A., M.S., etc.)	120 (31)	50 (29)
Bachelor’s degree (B.A., B.S.)	128 (33)	46 (26)
Associate’s degree	47 (12)	32 (18)
High school diploma or less	64 (16)	31 (18)
Prefer not to answer	3 (1)	2 (1)
Family Annual Income, *N* (%)		
$100,000 and over	115 (29)	47 (27)
$50,000 to $99,999	129 (33)	60 (34)
$30,000 to $49,999	70 (18)	32 (18)
Less than $29,999	57 (15)	20 (11)
Prefer not to answer	22 (6)	15 (9)
Relationship Status, *N* (%)		
Married or have a partner	202 (51)	94 (54)
Not married or have no partner	190 (48)	79 (45)
Prefer not to answer	1 (0.3)	1 (1)
Work Status, *N* (%)		
Retired	84 (21)	137 (79)
Not retired	307 (78)	35 (20)
Prefer not to answer	2 (1)	2 (1)

^1,2^ In the main analyses, ethnicity and race variables were recoded into a binary variable, where non-Hispanic White participants were coded as 0, while all others were coded as 1.

At Wave 1, the number of confirmed COVID-19 cases in this region surged, resulting in stricter physical distancing restrictions toward the end of data collection. Participants were recruited via phone or email with a brief description of the study. Inclusion criteria required that the participants resided in the local area (two counties in Southern California) during the study period, were fluent in English, had not been diagnosed with a cognitive condition (e.g., Alzheimer’s, dementia, or mild cognitive impairment), and had normal or corrected-to-normal eye vision. Once participants completed the informed consent, they were directed to a survey link on Qualtrics and responded to the questionnaire using their computer or mobile device. Except for one older adult who opted to have a research assistant aid them over the phone, all participants completed the survey independently. The first 100 participants enrolled in each age group received $20 as compensation. The remaining participants were informed that they would be included in the follow-up data collection with compensation.

### Measures

#### Growth mindset

To assess participants’ growth mindset, items from Dweck’s [35] Implicit Theories of Intelligence Questionnaire were used. Among 20 items in the original questionnaire, three items measuring growth mindset (e.g., “You can always substantially change how intelligent you are”) and three items measuring fixed mindset (e.g., “You can learn new things, but you can’t really change how intelligent you are”) were used. Participants indicated the degree to which they agree with each statement on a sliding scale from 1 = *Strongly disagree* to 7 = *Strongly agree*, by moving the slide marker to the desired point on the scale. Items assessing fixed mindsets were reverse coded, and an average was taken across the six items to form the composite. Higher scores represent a greater endorsement of growth mindset. In Study 1, growth mindset scores for one participant at Wave 1 and two participants at Wave 2 were treated as missing due to missing data on three or more items. In Study 2, the score of one participant at Wave 2 was treated as missing for the same reason. In the present study, the scale showed acceptable internal consistency, with Cronbach’s αs from Waves 1 to 3 being .73, .74, and .75 in Study 1 and .74, .74, and .73 in Study 2.

#### Depression

The Patient Health Questionnaire-9 (PHQ-9; [36]) was used to assess participants’ depression symptoms. This 9-item measure assesses symptoms of depression based on the criteria in the Diagnostic and Statistical Manual of Mental Disorders (DSM). Participants indicated how frequently they experienced the depression symptoms (e.g., “Feeling down, depressed, or hopeless”, “Feeling bad about yourself—or that you are a failure or have let yourself or your family down”) over the past two weeks on a sliding scale from 1 = *Not at all* to 10 = *Nearly every day*, by moving the slide marker to the desired point on the scale. A composite score was created using the average of the nine items, with higher scores representing more severe depression symptoms. In Study 1, seven participants at Wave 1 and two participants at Wave 2 did not have data on depression, as they did not respond to the entire item set. In Study 2, two participants at Wave 1 and two participants at Wave 2 had no data on depression scores. In the present study, the scale showed adequate internal consistency, with Cronbach’s αs from Waves 1 to 3 being .89, .90, and .88 in Study 1 and .86, .90, and .86 in Study 2.

#### Well-being

To assess participants’ well-being, the PERMA-Profiler [37] was used. This scale consists of 15 items assessing five components of well-being (PERMA: Positive emotion, Engagement, Relationship, Meaning, and Accomplishment) based on Seligman’s theory of well-being [38]. Additionally, this measure includes eight filler items assessing negative emotion, physical health, loneliness, and overall happiness. In the current research, we included 15 core items and one item measuring overall happiness in the analysis, resulting in 16 items in total. Participants indicated the degree to which they agree with each statement (e.g., “In general, to what extent do you lead a purposeful and meaningful life?”, “How often do you become absorbed in what you are doing?”) on a sliding scale from 1 = *Very low* to 7 = *Very high*, by moving the slide marker to the desired point on the scale. Average was taken across the 16 items to compute an overall well-being score. Higher scores represent greater overall well-being. In Study 1, three participants at Wave 1 and one participant at Wave 2 did not have scores for well-being, as they did not respond to the entire item set. In Study 2, three participants at Wave 1 had no well-being scores. In the present study, the scale showed adequate internal consistency, with Cronbach’s αs from Waves 1 to 3 being .93, .94, and .94 in Study 1 and .92, .93, and .93 in Study 2.

#### Adjustment of daily routines

To assess participants’ adjustment of daily routines during the pandemic, a single item was used: “How much have you been adjusting tasks in your daily routine to accommodate the physical distancing restrictions?” For this question, participants indicated their agreement using a sliding scale from 1 = *Not at all* to 10 = *A great deal* by moving the slide marker to the desired point on the scale. In Study 1, nine participants at Wave 1, seven participants at Wave 2, and two participants at Wave 3 did not respond to this question. In Study 2, four participants at Wave 1 and two participants at Wave 2 did not provide their responses.

### Analytic plan

To investigate the effect of growth mindset on three outcomes (i.e., depression, well-being, and adjustment), we employed three separate linear mixed-effects models. These models test both fixed effects (population-level averages) and random effects (subject-level variability) for each outcome. By including random effects, these models account for the dependence of repeated measurements on the same subject. In other words, random effects capture variability across different timepoints.

In each model, age, income, educational level, gender, race, relationship status, and work status were included as predictors alongside the focal predictor, growth mindset. In Study 2, which focused on older adults, intervention status was included as an additional predictor. For categorical predictors, the reference groups were 1) gender = male, 2) race = non-Hispanic White, 3) relationship status = not married or have no partner, 4) work status = retired, and 5) intervention status = control group. Additionally, time was included as a categorical predictor, allowing changes in outcome variables between timepoints to vary, with Wave 1 serving as the reference group. An additional Wald test for the time variable was conducted to test the difference between Wave 2 and Wave 3.

In all models, we tested the assumption of normality (i.e., that the residuals from the model should follow a normal distribution). In cases where this assumption was violated, scores of the outcome variable were log-transformed. For each model, all possible highest levels of interaction terms between every predictor were initially included, and we systematically removed predictors to find the optimal model based on the Bayesian Information Criterion (BIC) and likelihood ratio tests. Only significant interaction effects were retained in the final models presented herein. In testing main hypotheses, we used the NLME package [39] in the R software [40] to perform linear mixed-effects modeling. Descriptive statistics and reliabilities were computed using the statistical software jamovi [41], which is based on R [40].

## Results

Descriptive statistics for the study variables in each study are displayed in Table 2.

**Table 2:** Descriptive statistics for the study variables in Studies 1 and 2.

	Study 1							
	Growth Mindset *M* (*SD*)		Depression *M* (*SD*)		Well-Being *M* (*SD*)		Adjustment *M* (*SD*)	
Wave 1 (*n* = 392)	5.34 (1.12)^a^		3.44 (1.92)^a^		5.27 (1.03)^a^		5.64 (2.84)^a^	
Wave 2 (*n* = 191)	5.29 (1.12)^a^		3.26 (1.88)^a^		4.98 (1.13)^a^		5.78 (2.98)^a^	
Wave 3 (*n* = 135)	5.36 (1.13)^a^		3.12 (1.70)^a^		5.22 (1.07)^a^		3.68 (2.54)^b^	
	Study 2							
	Growth Mindset *M* (*SD*)		Depression *M* (*SD*)		Well-Being *M* (*SD*)		Adjustment *M* (*SD*)	
	INT	CTRL	INT	CTRL	INT	CTRL	INT	CTRL
Wave 1 (*n* = 165)	5.77^a^ (0.89)	5.08^a^ (1.14)	2.52^a^ (1.49)	2.67^a^ (1.50)	5.47^a^ (1.00)	5.51^a^ (0.81)	5.60^a^ (3.04)	5.18^ab^ (2.86)
Wave 2 (*n* = 89)	5.54^a^ (0.97)	4.95^a^ (1.18)	2.19^a^ (1.29)	2.63^a^ (1.70)	5.64^a^ (0.90)	5.23^b^ (0.95)	4.88^a^ (2.52)	4.77^a^ (2.84)
Wave 3 (*n* = 68)	5.36^a^ (0.97)	5.20^a^ (1.21)	2.45^a^ (1.53)	2.36^a^ (1.26)	5.60^a^ (0.98)	5.54^ab^ (0.77)	3.20^b^ (2.55)	3.56^b^ (2.44)

INT = intervention group; CTRL = control group; Possible ranges for each score are: 1 to 7 for growth mindset, 1 to 10 for depression, 1 to 7 for well-being, and 1 to 10 for adjustment of daily routines. Means in the same column with different superscript letters indicate statistical differences across timepoints in repeated-measured ANOVA after Holm correction to account for multiple comparisons (*p* < .05).

### Study 1

Results from Study 1 (younger to older adults) are presented in Table 3. For depression, older age was associated with lower depression, and females reported marginally lower depression than males. Participants reported marginally lower depression scores at Wave 2 than at Wave 1. Differences between Waves 1 vs. 3 and Waves 2 vs. 3 (*p* = .744 based on Wald test) were not statistically significant. Growth mindset emerged as a significant factor, such that as the growth mindset score increased by 1-unit, the depression score was estimated to decrease by 0.30-unit. Race and relationship status were not significant predictors of depression.

**Table 3:** Linear mixed-effects models for all outcome measures in entire sample.

Outcome	Predictor	Estimate	*SE*	*df*	*p*
Depression	Age	-0.04	0.01	288	< .001*
	Gender^1^	-0.31	0.18	367	.096^†^
	Wave^2^				
	2	-0.20	0.11	288	.074^†^
	3	-0.15	0.13	288	.230
	Race^3^	-0.05	0.17	288	.782
	Relationship Status^4^	-0.19	0.16	288	.219
	Growth Mindset	-0.30	0.06	288	< .001*
Well-Being	Age	0.01	0.003	288	.003*
	Gender^1^	0.40	0.11	372	< .001*
	Wave^2^				
	2	-0.18	0.05	288	< .001*
	3	-0.06	0.06	288	.261
	Race^3^	0.02	0.09	288	.837
	Relationship Status^4^	0.23	0.08	288	.006*
	Growth Mindset	0.23	0.03	288	< .001*
Behavioral Adjustment	Age	-0.02	0.01	279	.005*
	Gender^1^	0.07	0.28	369	.800
	Wave^2^				
	2	0.10	0.22	279	.670
	3	-2.04	0.26	279	< .001*
	Race^3^	-0.60	0.26	279	.023*
	Relationship Status^4^	0.30	0.25	279	.243
	Growth Mindset	0.28	0.11	279	.008*

Reference groups are ^1^ Male

^2^ Wave 1

^3^ Non-Hispanic White

^4^ Not married or have no partner.

*p < .05.

^†^*p* < .10.

For well-being, older age predicted higher well-being, and females reported higher levels of well-being. Participants who were married or had partners were estimated to have higher well-being scores than those who were not in relationships. Well-being scores were lower at Wave 2 than Wave 1, and an additional Wald test indicated that they marginally increased from Wave 2 to Wave 3 (*p* = .051 based on Wald test). Growth mindset was positively associated with well-being scores, such that as the growth mindset score increases by 1-unit, the well-being score was estimated to increase by 0.23-unit. Race did not predict participants’ well-being.

For adjustment of behaviors, age and race were significant predictors, such that the older the participants were, the lower levels of adjustment they showed, and non-Hispanic White participants reported higher adjustment of behaviors compared to participants who identified themselves as people of color (POC) or other. Participants also showed higher adjustment scores at Wave 1 than Wave 3, and higher at Wave 2 than Wave 3 (*p* < .001 based on Wald test). Growth mindset emerged as a significant predictor, predicting 0.28-unit higher adjustment scores per 1-unit increase. There was no significant difference in participants’ adjustment of behaviors by gender and relationship status.

### Study 2

Results from Study 2 (only older adults who were or were not in the intervention) are presented in Table 4. For this set of analyses, depression and adjustment of behavior scores were log-transformed, as the normality assumption was not satisfied for these variables. For depression, age and work status were significant predictors, such that older age was associated with decreased depression, and those who were not retired showed lower depression scores than those who retired. Those who were married or had a partner showed marginally lower depression scores compared to those not in a relationship. Other demographic factors (i.e., gender, race), intervention status, and time were not significantly related to depression. Per 1-unit increase in growth mindset, older adults reported approximately 9% lower depression scores.

**Table 4:** Linear mixed-effects models for all outcome measures in older adults.

Outcome	Predictor	Estimate	*SE*	*df*	*p*
Depression	Age	-0.02	0.01	137	.005*
	Gender^1^	-0.11	0.09	173	.255
	Intervention^2^	-0.04	0.08	173	.661
	Wave^3^				
	2	-0.06	0.04	137	.131
	3	-0.01	0.05	137	.808
	Race^4^	0.02	0.07	137	.807
	Relationship Status^5^	-0.12	0.07	137	.093^†^
	Work Status^6^	-0.19	0.10	137	.049*
	Growth Mindset	-0.092	0.03	137	< .001*
Well-Being	Age	0.01	0.01	134	.488
	Gender^1^	0.28	0.15	174	.074^†^
	Intervention^2^	-0.97	0.50	174	.054^†^
	Wave^3^				
	2	-0.28	0.09	134	.001*
	3	-0.09	0.10	134	.376
	Race^4^	0.05	0.12	134	.657
	Relationship Status^5^	0.38	0.11	134	.001*
	Work Status^6^	-0.002	0.15	134	.988
	Growth Mindset	0.19	0.05	134	< .001*
	Intervention × Growth Mindset	0.15	0.09	134	.087^†^
	Wave 2 × Intervention	0.36	0.14	134	.009*
	Wave 3 × Intervention	0.28	0.15	134	.062^†^
Behavioral Adjustment	Age	-0.02	0.01	133	.012*
	Gender^1^	0.14	0.11	175	.203
	Intervention^2^	0.03	0.10	175	.759
	Wave^3^				
	2	-0.09	0.08	133	.282
	3	-0.58	0.09	133	< .001*
	Race^4^	-0.20	0.09	133	.031*
	Relationship Status^5^	-0.01	0.09	133	.907
	Work Status^6^	-0.05	0.12	133	.680
	Growth Mindset	0.09	0.04	133	.022*

Reference groups are ^1^ Male

^2^ Control group

^3^ Wave 1

^4^ Non-Hispanic White

^5^ Not married or have no partner

^6^ Retired.

*p < .05.

^†^*p* < .10.

For well-being, female participants reported marginally higher well-being scores than males, and those who were married or had a partner showed higher levels of well-being than those not in a relationship. Age, race, and work status were not significantly associated with well-being scores. While intervention status, time, and growth mindset showed some associations with well-being, their interaction effects also emerged. Specifically, intervention status and growth mindset showed a marginally significant interaction, such that for the intervention group, a 1-unit increase in growth mindset score was associated with 0.34-unit increase in well-being, while it predicted 0.19-unit increase for the control group. Additionally, intervention status and time showed interaction effects. For those who participated in the intervention, they showed increased well-being from Wave 1 to Wave 2 by 0.08-unit, while for the control group, it decreased by 0.28-unit. Moreover, between Waves 1 and 3, well-being scores showed a marginal increase by 0.19-unit among the intervention group, while it decreased by 0.09-unit in the control group. Further Wald tests comparing Waves 2 and 3 indicated that, whereas there is no difference between these timepoints for the intervention group (*p* = .339 based on Wald test), the control group showed a marginal increase from Waves 2 to 3 by 0.19-unit (*p* = .075 based on Wald test). Keeping all the other predictors fixed, including the growth mind scores, the control group was estimated to show higher well-being by 0.82-unit (i.e., -0.97 + 0.15) at Wave 1. However, at Waves 2 and 3, this difference became smaller, 0.46-unit (i.e., -0.97 + 0.15 + 0.36) and 0.54-unit (i.e., -0.97 + 0.15 + 0.28), respectively, as the intervention group improved their well-being scores over time, while the control group’s scores decreased.

For adjustment of behaviors, older age was associated with lower adjustment, and participants showed higher adjustment scores at Wave 1 than Wave 3, and higher at Wave 2 than Wave 3 (*p* < .001 based on Wald test). Similar to Study 1, non-Hispanic White (vs. POC) participants reported higher adjustment of behaviors. Other demographic factors (i.e., gender, relationship status, work status) and intervention status were not associated with adjustment of behaviors. Growth mindset emerged as a significant predictor, predicting approximately 9% higher adjustment scores per its 1-unit increase.

## Discussion

The present study investigated whether endorsing a growth mindset (i.e., a belief that intelligence and abilities are malleable) would be associated with less depression, greater well-being, and adjustment during the COVID-19 pandemic. Utilizing a diverse sample of adults in the US, we found that while growth mindset was predictive of variabilities in mental health overall, the effects differed across sociodemographic groups. Analyses focusing on the older adults group showed that a learning intervention incorporating growth mindset prior to the pandemic may have produced some buffering effects. Our focal research question centered around the role of growth mindset in mental health outcomes over the course of the pandemic. Supporting the idea that espousing a growth mindset is conducive to mental health in general [42], we found that growth mindset is associated with adults’ lower levels of depression, higher well-being, and greater likelihood of adjustment of their daily tasks amid the pandemic. This finding adds to the extant evidence that growth mindset relates to better mental health [17] and coping when facing challenges, beyond its documented benefits on academic achievement [15, 24].

Our findings also shed light on the trajectories of mental health outcomes amid the pandemic in general, as well as age effects. Consistent with prior research reporting overall resilience in mental health over the course of the pandemic [43], adults in our Study 1 showed that although well-being scores declined from Wave 1 to Wave 2, there was some increase, approaching baseline levels by Wave 3. Given that adjustment of routine was higher at Waves 1 and 2 than Wave 3, our study also indicates that the need to adapt to the “new normal” might have been the greatest at the earlier stages of the pandemic. Interestingly, we also found the main effects of age, such that age predicted lower levels of depression and higher levels of subjective well-being. Some researchers argue that older adults may have faced additional stressors during the pandemic, such as an increased risk of social exclusion due to their lower use of digital technology [28]. However, evidence has found that older adults reported greater emotional well-being (i.e., more positive and less negative emotions; [44]) and less depressive and anxiety symptoms [45, 46], pandemic-related stress, and social isolation [47] than younger adults. Our findings echo prior research suggesting that although this pandemic may have posed a greater and unique risk of social disconnectedness and isolation for some older adults [26, 47], in general, older adults may be relatively resilient [43, 44]. However, older age was associated with lower adjustment of daily routines. In contrast to older adults, younger adults may have reported higher depression and lower well-being due to the necessity to adjust more to sudden changes related to the pandemic. For example, Birditt et al. [47] found that younger (rather than older) adults reported greater levels of pandemic-related life changes, which were associated with poorer well-being.

Study 2 focused on older adults, with special attention to the effects of a learning intervention prior to the pandemic on mental health of older adults. Consistent with Study 1, we found that older adults showed the greatest levels of adjustment of daily routines during the initial phase of the pandemic. In addition, growth mindset was associated with lower levels of depression and greater adjustment of daily routine. For subjective well-being, however, growth mindset and time showed varied effects between the intervention vs. control groups. When probing the marginally significant interaction effects between the intervention status and growth mindset, we found that growth mindset was associated with an increase in well-being to a greater extent in the intervention (vs. control) group. Another notable finding was an interplay between time and intervention status on subjective well-being, such that whereas the intervention group showed an increase in subjective well-being over time, it decreased among the control group.

Recalling the prior intervention study [30] where older adults in the intervention showed similar patterns in cognitive functioning of younger adults, our findings suggest that these participants may be similar to younger adults in terms of subjective well-being as well. Although older adults in the intervention group fared worse in terms of overall levels of well-being than those in the control group at the initial timepoint, the interaction effect revealed that the conducive role of growth mindset in this outcome was greater in the intervention group. This finding indicates that older adults in the intervention group may have experienced lower levels of well-being during the initial phase of the COVID-19 pandemic, but they showed more resilience in subjective well-being compared to the control group. Moreover, the buffering effect of growth mindset may have been more salient among these individuals. As such, this finding indicates that the potential buffering effect of growth mindset on older adults’ well-being was largest when accompanied by cognitively stimulating activities and learning experiences provided before the pandemic.

## Limitations and future directions

Several limitations to the current study should be noted. First, the sample included in our study does not represent the population of the US. In particular, the majority of our sample consisted of females, and our study could only include individuals with access to the Internet, given its online administration. Second, we only assessed growth mindset as traditionally defined (i.e., beliefs about the malleability of intellectual abilities). Although extant literature suggests that growth mindset about intelligence is related to mental health [17, 18], there can be other domain-specific beliefs, such as mindsets of emotions [42, 48] or perceived capability of emotion regulation or coping [49], that may be more closely related to mental health. Hence, future research evaluating the role of emotion- or mental health-related or beliefs in mental health may be beneficial. Lastly, for Study 2, although we speculate that prior success experiences (the behavioral component) interplayed with growth mindset (the cognitive component) to produce a synergetic effect on mental health, we were limited in directly testing this idea. For example, it may be the case that growth mindset fosters older adults’ actual involvement in learning new skills or enhanced emotional regulation [16, 48], which in turn, predicts better mental health.

## Conclusion

The present study enhances our understanding of the relationship between growth mindset, depression, well-being, and adjustment in American adults amidst the COVID-19 pandemic. Consistent with the postulation in prior research, motivational factors including growth mindset may play a beneficial role in individuals’ mental health in the face of challenges. Given that growth mindset is a cognitive belief that can be taught, incorporating growth mindset approaches into interventions in response to the pandemic might be fruitful. Our findings also suggest the potential synergetic interplay between prior learning intervention experience and growth mindset on mental health. These results may be informative for developing future interventions to promote mental health and adjustment during a pandemic and in its aftermath, as well as in other challenging situations when individuals face changing life circumstances.
